# Rapid cold plasma synthesis of cobalt metal–organic framework/reduced graphene oxide nanocomposites for use as supercapacitor electrodes

**DOI:** 10.1038/s41598-023-41816-9

**Published:** 2023-09-13

**Authors:** Zeinab Karimzadeh, Babak Shokri, Ali Morsali

**Affiliations:** 1 Laser and Plasma Research Institute, Shahid Beheshti University, P.O. Box 1983969411, Tehran, Iran; 2 Faculty of Physics, Shahid Beheshti University, P.O. Box 1983969411, Tehran, Iran; 3 Department of Chemistry, Faculty of Sciences, Tarbiat Modares University, P.O. Box 14115-175, Tehran, Iran

**Keywords:** Energy science and technology, Materials science, Nanoscience and technology

## Abstract

Metal–organic frameworks (MOFs) are recognized as a desirable class of porous materials for energy storage applications, despite their limited conductivity. In the present study, Co-MOF-71 was fabricated as a high-performance supercapacitor electrode at ambient temperature using a fast and straightforward, one-pot cold plasma method. A supercapacitor electrode based on Co-MOF@rGO was also synthesized by adding reduced graphene oxide (rGO) during processing to increase the capacitance retention and stability after 4000 cycles from 80 to 95.4%. The Co-MOF-71 electrode provided a specific capacitance (Cs) of 651.7 Fg^−1^ at 1 Ag^−1^, whereas the Co-MOF@rGO electrode produced a Cs value of 967.68 Fg^−1^ at 1 Ag^−1^. In addition, we fabricated an asymmetric device (Co-MOF@rGO||AC) using Co-MOF-rGO as a high-rate positive electrode and activated carbon (AC) as a negative electrode. This hybrid device has a remarkable specific energy and power density. The combination of MOFs with reduced graphene oxide (rGO) in a cold plasma environment resulted in the formation of a three-dimensional nanostructure composed of nanosheets. This nanostructure exhibited an increased number of electroactive sites, providing benefits for energy storage applications.

## Introduction

The demand for sustainable and efficient energy storage systems has increased considerably due to the extensive use of fossil fuels and rising population growth. Thus, high-performance energy storage devices, such as batteries and supercapacitors, are receiving more attention. Supercapacitors exhibit considerable potential as a viable alternative owing to their enhanced power density, accelerated charge and discharge capabilities, prolonged operational lifespan, and diminished mass. However, their low energy output limits their usefulness, so new electrode materials were required^1–3^. One strategy is to develop and identify novel materials, such as metal–organic frameworks (MOFs), that exhibit both electrochemical double-layer storage (EDLC) and rapid surface redox reactions^4–6^.

Due to inherent properties such as structural flexibility, crystalline nature, and a large specific surface area, metal–organic frameworks have been excellent candidates not only for electrochemical energy storage^7,8^ but also in applications such as gas storage and filtration^9,10^, sensors^11^, catalysis^12–14^, and drug delivery^15–17^. The direct application of a metal–organic framework (MOF) as an electrode material has the potential to reduce energy consumption and costs related to the commercialization of MOF-based energy storage technologies. However, previous research on Fe-MOFs^18^, Ni-MOFs^19^, Co-MOFs^20^, and Zn-MOFs^21^ revealed that pristine MOFs are not appropriate for energy storage because of their limited conductivity and energy density.

Numerous techniques have been proposed for solving this issue^22^. Combining MOFs with highly conductive materials such as metals^23,24^, carbon nanotubes^25,26^, graphene^27,28^, or conductive polymers^29,30^ is the most common method. Using these hybride materials is useful due to their increased intrinsic conductivity and unique structures. The work undertaken by Kim et al. provides an illustration of this phenomenon, wherein they synthesised a hybrid material comprising Sn–Co–S and MXene^31^. This hybrid material exhibited a specific capacity of 305.71 mA h gm^−1^ at a current density of 1 Ag^−1^. A cathode material with an areal capacity value of 1.43 mA h cm^−2^ at 1 mA cm^−2^ was created by Alam et al. using MOF-driven NiS@Co-Mo-LDHs shell@core^32^. In their study, Alam et al. presented the synthesis of novel anode materials for asymmetric supercapacitors, which consisted of silver nanoparticles embedded within a titanium carbide (Ti_3_C_2_) matrix and were fabricated using a 0D@2D printing technique^33^. The resulting anode material demonstrated a specific capacitance of 368.56 Fg^−1^ when tested at a current density of 1 Ag^−1^. Alam et al. used an ink-printed electrode made of ${\text{TiO}}_{2}$ and Ag nanoparticles^34^, which had a maximum specific capacitance of 257.8 (122.96 mA h mg^−1^) Fg^−1^ at 1 Ag^−1^. The study conducted by Saeed and colleagues involved the presentation of the synthesis and characterization of nanosheet arrays consisting of manganese-cobalt-sulphur (Mn-1) CoxSy^35^. These nanosheets are enveloped by distinctive marigold flower-like nanoreservoirs composed of nickel-copper hydroxides (Ni-CuOHs). In another study, Alam et al. integrated boron nitride nanotube (BNNT) powder into a binary composite material consisting of activated carbon (AC) and multi-walled carbon nanotubes (MWCNTs) with the aim of improving the electrochemical properties of the composite^36^. Furthermore, according to Hao et al., combining MXenes (Ti_3_C_2_ T_x_) and ZIF-8 can improve the performance of the hydrogen evolution reaction (HER)^37^. Hussain and his colleagues have used a solvothermal method to make an ethanol sensor out of 2D WSe_2_ nanosheets (NSs) and a WSe_2_/rGO hybrid material^38^. They use rGO that is wound on WSe_2_ NSs to improve electron transmission across the NSs in their research. In their study, Hanan et al. investigated the use of reduced graphene oxide (rGO) combined with iron oxide (Fe_3_O_4_) nanostructures as an electrocatalyst with high activity for the hydrogen evolution reaction in alkaline media^39^. The overall electrochemical measurements of rGO@Fe_3_O_4_ have been enhanced due to the improved conductivity and the introduction of different ionic charges by dissimilar elements within crystal lattices. Moreover, Hanan et al. synthesized composite systems comprising cobalt ferrite oxide and reduced graphene oxide (Co_2_Fe O_4_@rGO) for the purpose of trifunctional water splitting^40^. The incorporation of reduced graphene oxide (rGO) into the composite offers several benefits. rGO’s exceptional electrical conductivity and large surface area facilitate rapid electron transport, improving charge transfer kinetics and reducing internal resistance. It also facilitates ion diffusion and accessibility, reducing ion transport limitations and enhancing overall charge storage capacity. Additionally, rGO plays a pivotal role in extending the composite’s long-term cycling stability by mitigating structural degradation and particle agglomeration during repeated charge–discharge cycles. Furthermore, rGO can promote and enhance faradaic contributions to the overall capacitance, facilitating charge transfer processes associated with specific redox-active centers within the MOF structure. These collective benefits synergistically enhance the electrochemical performance of the composite, justifying its incorporation as a means to achieve superior supercapacitor characteristics compared to standalone MOF electrodes^41,42^. Nevertheless, there have been obstacles, such as the difficulty of combining materials uniformly, the complexity of the preparation process, and the need for high temperatures and extended periods in the composition process^43^. Consequently, additional research is required to develop effective methods for MOF composite production and direct use in energy storage devices.

Furthermore, the electrochemical efficacy of electrodes is dependent not only on the type and content of the electrode but also on its morphology, porosity, and dimension. The performance of supercapacitors can also be enhanced by constructing a unique architecture that allows extensive use of nanoporous structural features. In general, morphological modification has a considerable effect on the functioning of active material. Because of their distinct pore size distribution and higher surface area, nanoporous materials exhibit superior electrochemical properties compared to bulk materials. Such characteristics facilitate electrolyte diffusion and ion transport. Even though there are problems with chemical agents, adding chemical components like surfactants or counter-ions to the reactants is a common way for standard protocols to control the shape of MOFs. These additives impede the swift development of the MOF nucleation phase^43–45^.

On the other hand, conventional methods of synthesis, such as hydrothermal or microwave synthesis, typically demand energy, cost, a longer reaction time, or the use of complicated processes and special chemicals. As a consequence, the search for breakthrough techniques and materials with optimal structures remains a major priority for future electrochemical energy storage systems. Recently, a new approach based on cold plasma has been reported as a solution to these issues. Compared to conventional preparation methods (such as hydrothermal, which often require several hours or even days and heating equipment), cold plasma has significant advantages, including ease, energy savings, and high synthesis speed. Therefore, it can generate more active sites with distinct structures and morphologies without using any chemicals^46–50^. Among the various plasma sources, dielectric barrier discharge (DBD) possesses the properties of a non-equilibrium cold plasma, which is characterised by a comparatively low macroscopic temperature and a high electron temperature. The perturbation of plasma equilibrium has a critical role in facilitating chemical reactions. Although the energy of electrons can successfully ignite relatively stable (or even neutral) small molecules to start a chemical reaction, the entire reaction system was kept at a low temperature for industrial applications. The production of DBD plasma is considered to be a relatively straightforward and cost-efficient procedure, as it can be achieved at standard room temperature and atmospheric pressure, eliminating the need for a vacuum environment. In addition, the utilisation of plasma synthesis obviates the necessity for surfactants, hazardous reducing agents, stabilisers, and specialised solvents, commonly employed in the fabrication of metal nanoparticles and metal oxides. For the production of MOF nanoparticles, this method is reliable, inexpensive, environmentally friendly, and quick^51,52^.

This study examined the effect of simple one-step plasma synthesis on the structural, physical, and electrochemical properties of the Co-MOF supercapacitor electrode system. In addition, the specific capacitance, and capacity retention of supercapacitors are measured and discussed. Second, the effect of adding rGO during the synthesis of the Co-MOF-rGO supercapacitor electrode on its electrochemical stability and specific capacitance is investigated. The Cs of Co-MOF-rGO were determined to be 967.68 Fg^−1^ at 1 Ag^−1^, higher than Co-MOF (651.7 Fg^−1^). After 4000 cycles, the Cs retention of this electrode exhibited excellent 95.4% stability. In addition, we constructed an asymmetrical device by employing Co-MOF-rGO and activated carbon (AC) as the positive and negative electrodes, respectively, in the configuration of Co-MOF@rGO||AC. The hybrid device exhibits a noteworthy specific energy value of 41.82 W ${\text{h}}\;{\text{kg}}^{ - 1}$ and a power density of 284 W ${\text{kg}}^{ - 1}$ at a current of 1 A${\text{g}}^{ - 1}$. Generally, plasma leads to the formation of a 3D nanosheet morphology that enhances electrochemical performance.

## Methods

### Materials

Potassium hydroxide (KOH), Cobalt (II) nitrate hexahydrate (${\text{Co}}({\text{NO}}_{3} )_{2}$* 6 ${\text{H}}_{2} {\text{O}})$, nickel (II) nitrate hexahydrate (${\text{Ni}}({\text{NO}}_{3} )_{2}$*6 ${\text{H}}_{2} {\text{O}}$), terephthalic acid (${\text{C}}_{8} {\text{H}}_{6} {\text{O}}_{4}$), N, N-dimethyl formamide (DMF), and ethanol were purchased from Merck. Reduced graphene oxide (rGO) was bought from Borhan Co., Iran. No additional processing was required, and all chemical compounds were utilized as supplied.

### Synthesis of pristine MOF

A homemade DBD-based reactor is used in this work for MOF preparation. All MOFs were produced by straightforward, one-step plasma synthesis. Firstly, 1 mM (0.291 g) ${\text{Co}}({\text{NO}}_{3} )_{2}$* 6 ${\text{H}}_{2} {\text{O}}$ and 1 mM (0.166 g) terephthalic acid (BDC) were mixed in 12 ml of DMF. After that, the combined solution was stirred for 15 min while 1.8 ml of ethanol was added drop by drop to create a homogeneous mixture. Following that, the solution was transferred to the DBD plasma reactor, where it was discharged for 30, 45 and 60 min, respectively (Table S1). Then, the mixture was washed three times with DMF and ethanol. The purple substance was gathered and dried for 12 h.

### Synthesis of MOF@rGO composites

MOF@rGO nano-composites were made, and their ability to store energy was investigated in order to determine the influence of reduced graphene oxide as a conductive compound on the electrochemical properties of the MOF. Multiple weight percentages (wt%) of MOF:rGO (1:2, 1:1, and 2:1) were sonicated for 30 min in DMF with ${\text{Co}}({\text{No}}_{3} )_{2}$* 6 ${\text{H}}_{2} {\text{O}}$ to adjust the Co-MOF@rGO ratio in the nanocomposite for optimal supercapacitive behavior. The mixture was subsequently sent to the DBD reactor and discharged at 6.5 kV for 45 min. The resulting black solid was rinsed many times with DMF and ethanol before being dried for 12 h.

### Plasma reactor

**Figure 1:**
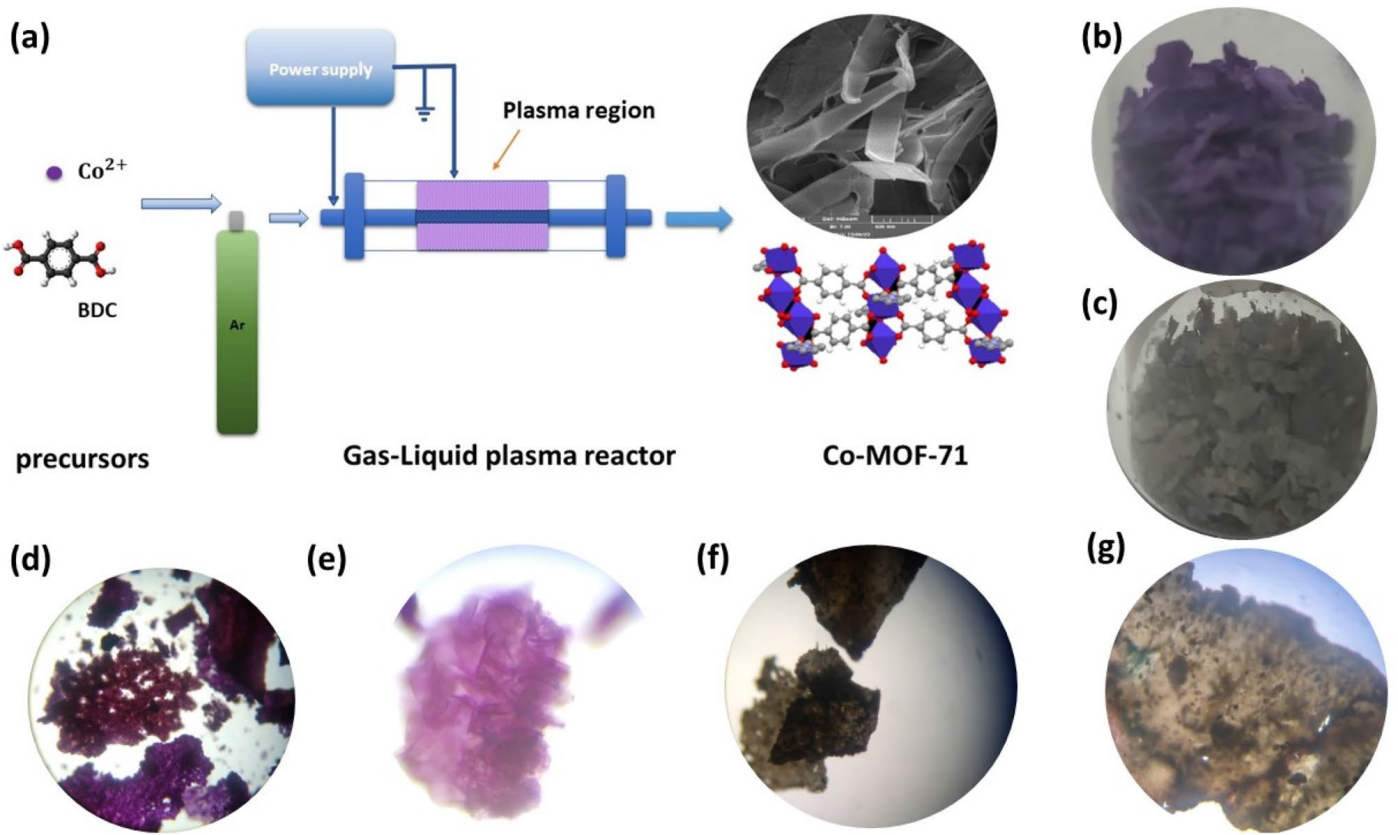
(**a**)A schematic diagram of the DBD gas–liquid cold plasma device for synthesizing MOF-71 and MOF composites. Synthesised (**b**) MOF-71, (**c**) Co-MOF-rGO. (**d**, **e**) optical image of Co-MOF in 10X and 40X resolution. (**f**, **g**) optical image of Co-MOF-rGO nanocomposite in 10X and 40X resolution.

Figure 1a depicts a schematic diagram of the DBD cold plasma device for synthesizing Co-MOF and single-step Co-MOF-rGO production at atmospheric pressure. The cylindrical DBD cold plasma reactor makes up for this purpose, which includes the stainless-steel mesh (200 mm long) that functions as a low-voltage electrode and the stainless-steel rod (300 mm long and 10 mm in diameter) that acts as a high-voltage electrode, along with a quartz tube (300 mm long and 14.5 mm inner diameter). A handmade AC power supply with a maximum output of 130 W at 14 kHz was used to turn on the plasma. A Tektronix (TDS-2024B, USA) oscilloscope and a Tektronix high-voltage probe (P6015A, USA) were used to record the discharge voltage. As the working gas, ultra-pure argon gas (> 99.999%) with a flow rate of 150 sccm (standard cubic centimeter per minute) was used. The discharge gap measured 4.5 mm. As indicated in Table S1, a planned investigation was conducted to optimize the synthesis parameters in terms of discharge time, discharge voltage, and reactant concentration. From now on, Co-MOF, and Co-MOF@rGO are defined as those that were produced under excellent synthesis conditions. Additionally, the optical images of these synthesised structures are depicted in Fig. 1b–g.

Further details regarding the fabrication of electrodes, the electrochemical techniques employed, calculations, and extra analyses can be found in the supplemental information section.

## Results and discussion

### Structural characterization

**Figure 2:**
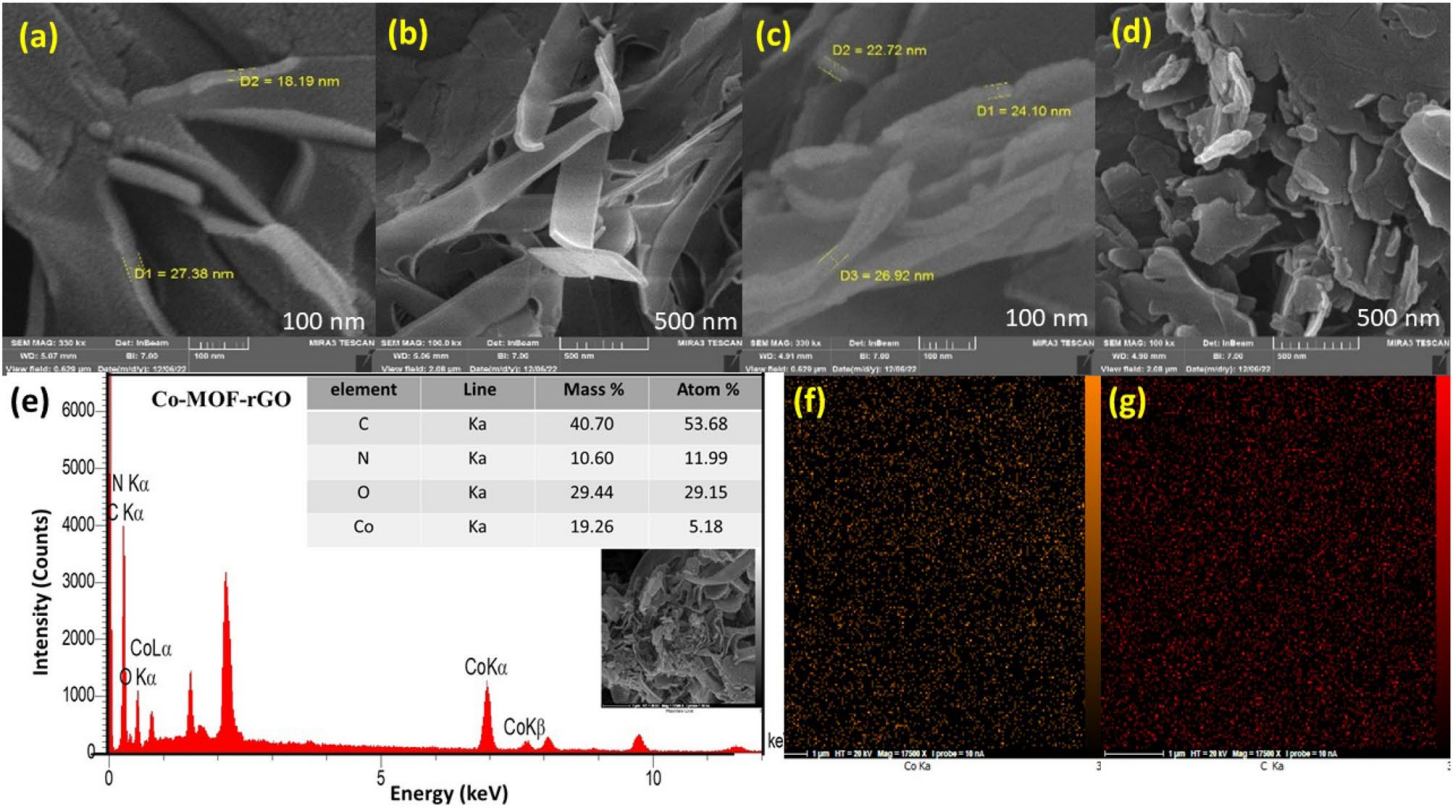
FE-SEM images of (**a**, **b**) Co-MOF, (**c**, **d**) Co-MOF@rGO nano-composite synthesized with plasma, respectively. (**e**) EDX spectrum of the as-synthesized Co-MOF@rGO, (**f**, **g**) EDS elemental mappings of cobalt and carbon elements of the Co-MOF@rGO nano-composite.

Field emission scanning electron microscopy (FESEM) was used to analyze the morphology of the generated MOFs. The Co-MOF morphology consists of a highly porous nanosheet array with an average thickness of about 20 nm (Fig. 2a, b). Clearly, the manipulation of plasma characteristics has the potential to exert a substantial influence on the structure of metal–organic frameworks (MOFs). In this study, it was seen that when the plasma voltage was raised, the regularity of layered Co-MOF nanosheets got better. Consequently, a highly porous three-dimensional network was produced, exhibiting characteristics that make it more suitable for use in supercapacitor applications. (Figure S4).

As shown in Figure S5, when rGO is introduced into the Co-MOF structure, it is positioned between and on the surface of the Co-MOF layers. By increasing the ratio of rGO to ${\text{Co}}({\text{NO}}_{3} )_{2}$* 6 $H_{2} O$, its network structure is obliterated, leading to a reduction in active redox sites. However, at the optimal ratio (Fig. 2c, d and S5b), the presence of rGO increases the structure’s conductivity and electrolyte diffusion by producing more conductive channels within the Co-MOF@rGO nanocomposite structure and improves its supercapacitor’s electrochemical performance (as we will explain in the electrochemical section). Compared to a previous report on MOF-71, it is crucial to note that gas–liquid plasma synthesis can alter the morphological structure and produce a more porous 3D nanostructure^53,54^.

Energy-dispersive X-ray spectroscopy (EDX) was used to examine the elemental composition and distribution of metal ions in the Co-MOF and Co-MOF@rGO nanocomposite (Fig. 2e and S6). The EDX and the EDX mapping images of the Co-MOF and Co-MOF@rGO samples (Fig. 2f, g and S7) confirmed the existence and uniform distribution of Co, C, and O atoms throughout the nanocomposite structure.

**Figure 3:**
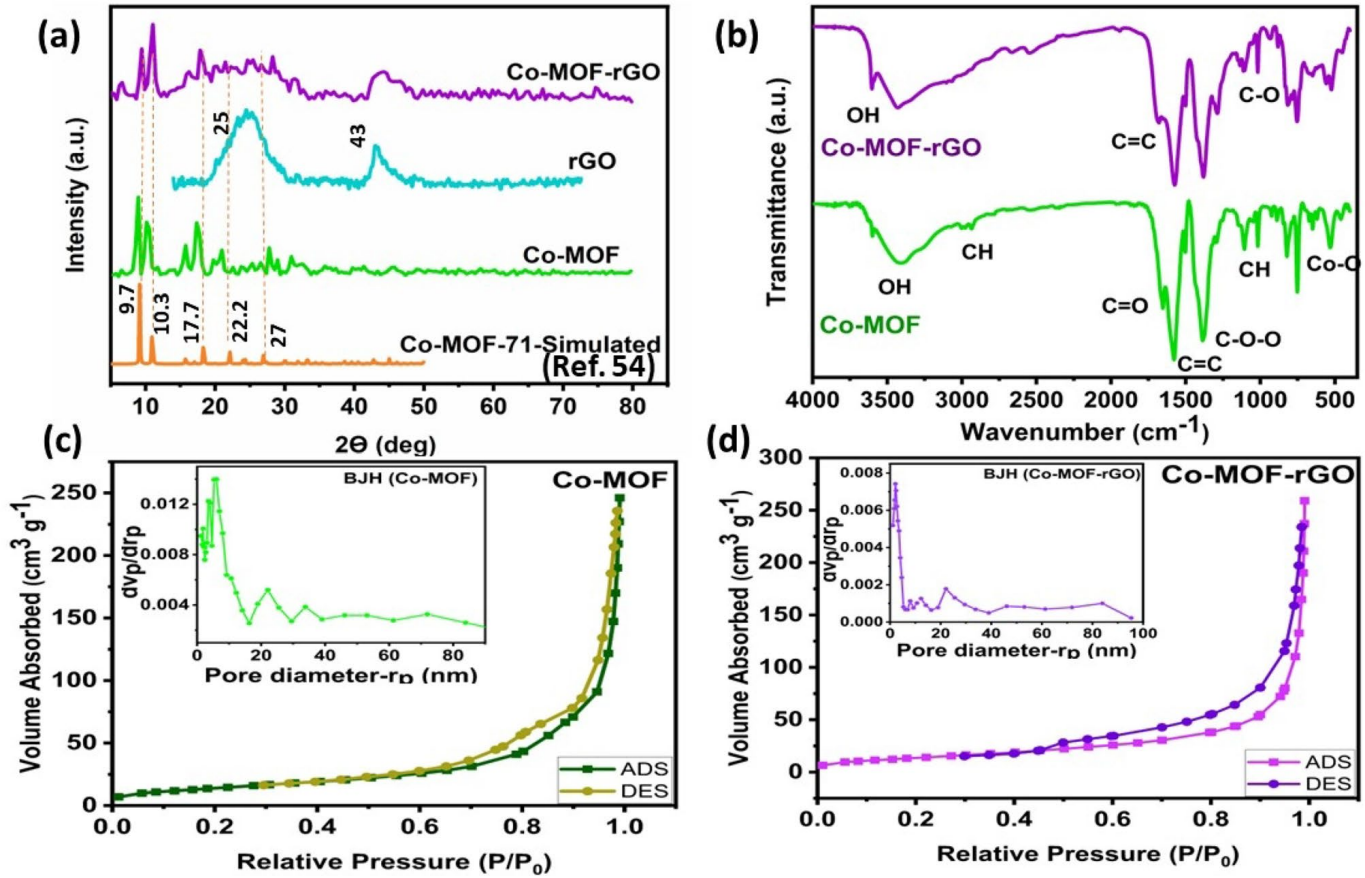
(**a**) XRD patterns of Co-MOF, rGO, Co-MOF@rGO composite and simulated MOF-71. (**b**) FT-IR spectra of Co-MOF, and Co-MOF@rGO composite. (**c**) BET and BJH analysis of Co-MOF. (**d**) BET and BJH analysis of Co-MOF-rGO.

The X-ray diffraction analysis (XRD) peaks for Co-MOFs are depicted in Fig. 3a, exhibiting peaks at approximately 9.7, 10.3, 15.6, 17.7, 22.2, and 27 degrees. The diffraction pattern exhibits a strong resemblance to the previously observed structure of MOF-71^55^. Sharp and narrow peaks with a consistent baseline suggest the highly crystalline structure of the MOFs. Figure S2 depicts the effect of preparation parameters, such as discharge duration and voltage, on the XRD peaks of the synthesis sample. Crystallinity increases with preparation time and voltage, whereas beyond a certain threshold, plasma no longer contributes to crystal structure. In general, the XRD peaks are sharper and stronger at the optimal voltage (peak to peak) of 6.5 kV and time of 45 min. Furthermore, the composite has a strong peak at 25 degrees and a weak peak at 43 degrees due to the (001) plane of graphene^56,57^. The similarity of other peaks to the purified sample indicates that rGO has no effect on the composite crystal structure.

The main FTIR (Fourier Transform Infrared Spectroscopy) bands of Co-MOF include the wide stretching band of OH (3428 ${\text{cm}}^{ - 1}$), the small wide stretching band of CH (2972 ${\text{cm}}^{ - 1}$), the small stretching band of C=O (1679 ${\text{cm}}^{ - 1}$), the vibrational double band of C=C (1579, 1510 ${\text{cm}}^{ - 1}$), the sharp vibrational band of C–O–O (1379 ${\text{cm}}^{ - 1}$), and three absorption bands (1094, 1010, and 808 ${\text{cm}}^{ - 1}$), which are the vibrational CH bands characteristic of the aromatic ring (Fig. 3b). Nevertheless, peak at approximately 557 ${\text{cm}}^{ - 1}$ is associated with the metal bonding between the metal atoms and the carboxylic group of the BDC ligand (Co–O band). In addition, the FTIR pattern of Co-MOF@rGO reveals the distinct absorption bands of rGO^58^, which correspond to the stronger and broader OH stretching band at about 3580 ${\text{cm}}^{ - 1}$, as well as the C=C vibrational peak at about 1665 ${\text{cm}}^{ - 1}$ and the C–O at about 1107 $cm^{ - 1}$.

The BET (Brunauer–Emmett–Teller) curve shows that Co-MOF and Co-MOF-rGO possesses a type IV nitrogen adsorption/desorption isotherm (Fig. 3c, d). The initial steep slope of the graph reveals micropores (< 2 nm), whereas the continuous increase in absorption with increasing relative pressure indicates monolayer or multilayer nitrogen molecule absorption in mesopores (2–50 nm). Absorption at increasing relative pressures (p/$p_{0}$ ~ 1) shows mesopore and micropore volumes. In addition, the layer structure can be inferred based on the diagram’s shape^59^. Overall, the gas–liquid plasma-produced Co-MOF has a larger surface area than earlier MOF-71 experiments (Table S3)^53^. Furthermore, the Barrett-Joyner-Halenda (BJH) technique demonstrated that the Co-MOF contained pores from 1 to 10 nm, with the largest being 6 nm while the Co-MOF-rGO nanocomposite had smaller pores (about 2.9 nm). The findings suggest that the incorporation of reduced graphene oxide (rGO) may lead to a reduction in the pore size of the structure, while yet allowing for unobstructed pore functionality.

**Figure 4:**
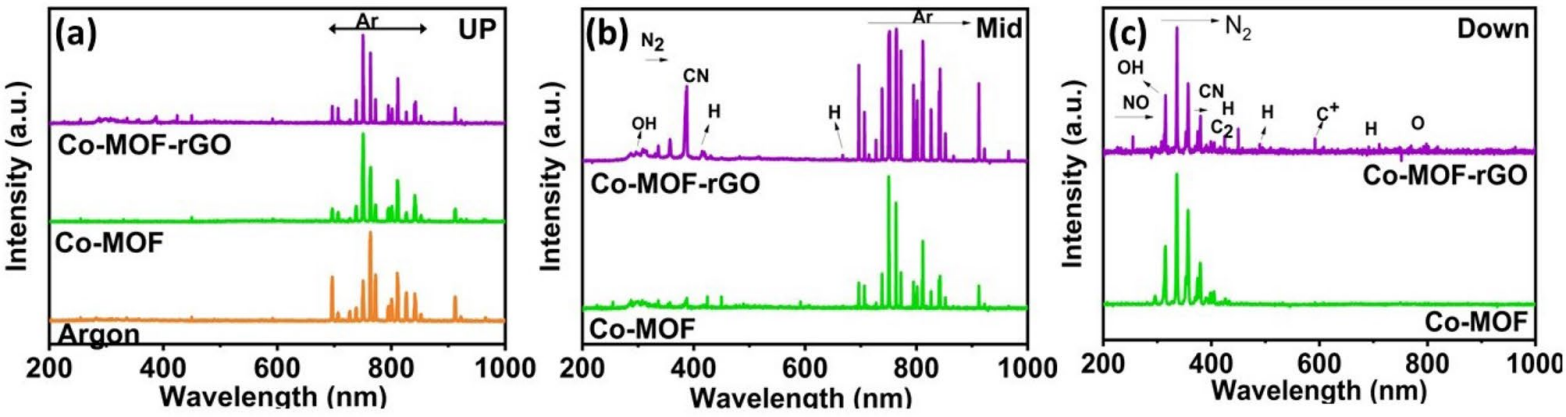
Optical emission spectra of plasma discharge in the (**a**) plasma gas (up), (**b**) interface (middle), and (**c**) plasma solution (down).

In order to comprehend how gas–liquid plasma generates MOF nanostructures, we analysed the optical emission spectrum (OES) at three locations within the reactor (Fig. 4). The uppermost, middle, and lowermost regions were located within the gaseous phase, the interface between the gas and liquid phases, and the liquid solution, respectively. The upper part featured spectral lines from 650 to 850 nm associated with argon working gas. The emission spectrum was produced when electrons collided with a high-energy carrier gas (Ar), causing gas atoms to transition from the ground state to the excited state and then to unstable lower energy states.

1 $${\text{Ar}} + e^{ - } \to {\text{Ar}}^{*} + e^{ - }$$

Within the intermediate region, argon lines are present alongside nitrogen and hydrogen peaks. In the solution, various spectral lines such as N2, NO, CH, and OH can be observed. Additionally, certain lines associated with $C^{ + } \;and\;C_{2}$ were identified in the Co-MOF@rGO sample^60–62^.

In general, plasma consists of electrons, positive and negative ions, atoms and molecules in their ground and excited states, reactive species, and a strong electric field. Radicals and electrons can enter the solution during plasma production, starting complex chain reactions. Thus, it may be challenging to perceive the processes of producing excited species and plasma dynamics on the emission spectrum. Strong electrons and free radicals accelerate chemical processes in plasma. High-energy electrons may attack the solution and cause chemical reactions with its ions. In addition, high-energy electrons travel faster than heavier ${\text{Ar}}^{ + }$ ions, providing a strong electric field. This electric field may generate a potential gradient at the plasma-liquid interface due to the separation of positive and negative ions. It quickly breaks up the positively charged metal cations $\left( {{\text{Co}}^{2 + } } \right)$ and ${\text{NO}}_{3}$ ions, facilitating the subsequent formation of coordination bonds.

On the other hand, organic linkers must be deprotonated before synthesis. This is usually done by heating or adding a Brønsted base (Brønsted–Lowry theory). Plasma can accomplish this task due to its strong electric field and active species. Therefore, electrons at the gas–liquid plasma interface can alter the pH of the solution. Achieving a balance between deprotonation and protonation via the electric field results in the further deprotonation of the linkers through the isolated proton^63^. Additionally, the solution contains hydrogen, according to OES. The electrons can easily separate this hydrogen from the linkers and turn it into hydride. Furthermore, the plasma OES spectrum shows CN species, which may result from the formation of an intermediate molecule that has a stable C–N bond. As Jiang et al. described, DMF radicals produced in the plasma field could be consumed to form intermediate DMC molecules^50^. In addition, plasma discharges contain OH radicals. Due to the electronegative nature of OH, cations can easily convert it to ${\text{OH}}^{ - }$ by giving it their electrons. Consequently, the metal hydroxide and organic linker form a coordination bond. This enables the crystal to grow from atoms, molecules, and ions layered on top of a nucleus. Further research will be conducted to investigate the impact of plasma variables on MOF properties.

### Electrochemical measurements

**Figure 5:**
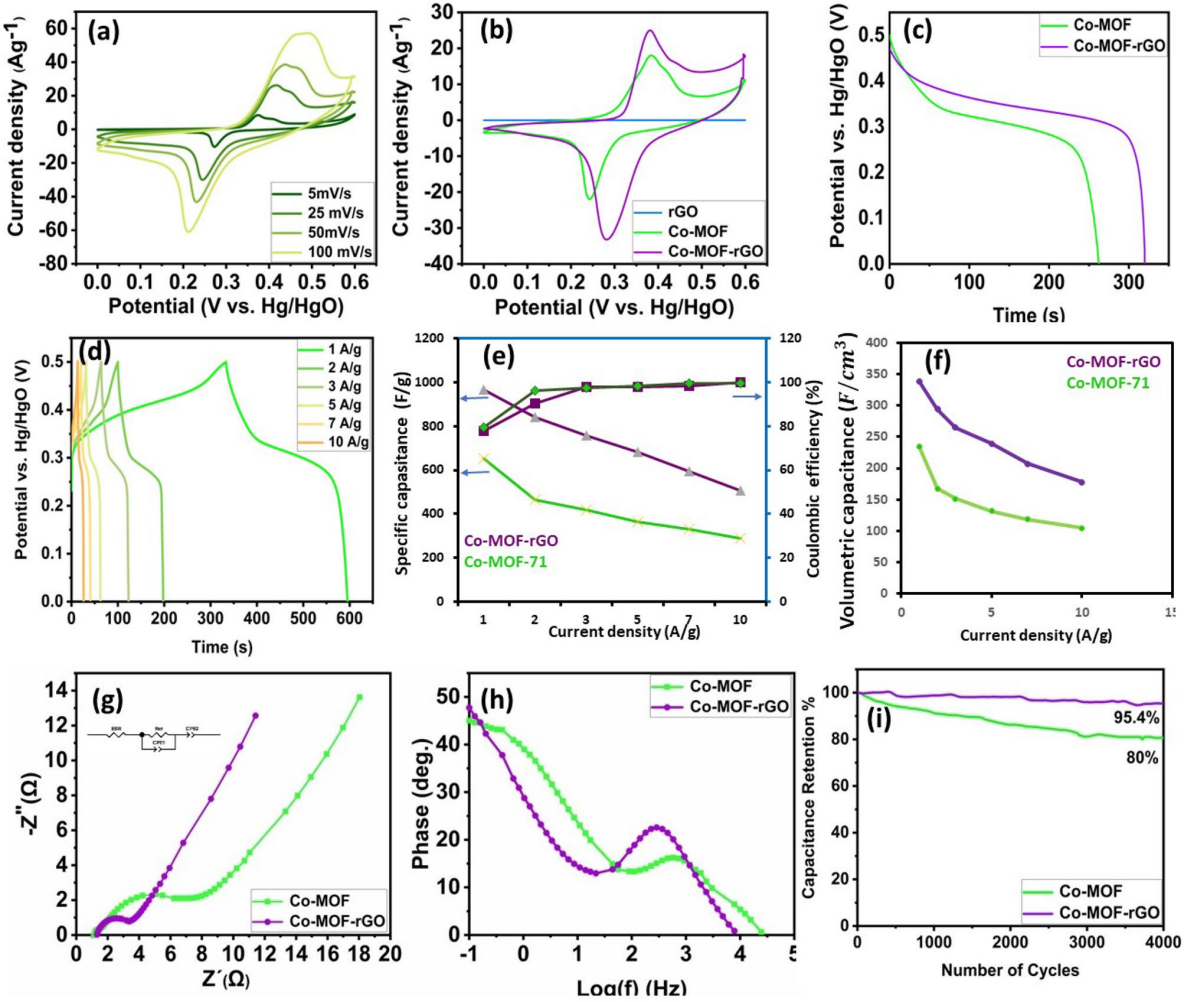
(**a**) CV curves of the Co-MOF electrode at different scan rates from 5 to 100 mV ${\text{s}}^{ - 1}$. (**b**) CV curves of Co-MOF, Co-MOF-rGO composite and rGO at 5 mV ${\text{s}}^{ - 1}$. (**c**) Discharge profiles of the electrodes at 1 A${\text{g}}^{ - 1}$. (**d**) Discharge profiles of Co-MOF at different current densities. (**e**) capacitance retention, and (**f**) coulombic efficiency for two nanomaterials. (**g**) Nyquist and (**h**) Bode phase plots of all the synthesized electrodes in the frequency range from 100 kHz to 0.1 Hz at an open-circuit potential, and (**i**) capacitance retention vs number of cycles of the two nanomaterials. All the electrochemical studies were performed in a 6.0 M KOH electrolyte.

Figure 5a displays Co-MOF electrode CVs (cyclic voltammetry) at varying scanning rates. At all scanning velocities, the cathodic and anodic redox peaks demonstrate that faradic reactions dominate charge storage in active materials. At slower scan rates, MOF reacts sequentially and reversibly with dissolved anions at the electrode/electrolyte interface. This produces two oxidation peaks and one reduction peak. Although the scan rate increases the intensity of the redox peak, this indicates rapid Faraday reactions on the surface of the electrode material and low resistance. However, efficiency is diminished because ions cannot reach distant micropores in active materials at high scan velocities.

The closed area of the cyclic voltammetry (CV) curve serves as an indicator of the electrode’s capacity. In order to optimise the supercapacitive properties of the nanocomposite, thereby facilitating improved charge storage capacity, efficient electron transport, and greater electrolyte accessibility, we conducted an analysis of three different ratios of metal–organic framework (MOF) to reduced graphene oxide (rGO) (1:2, 1:1, and 2:1) using cyclic voltammetry (CV) curves. The chosen ratios facilitate the clarification of the impact of MOF and rGO on the electrochemical activity of the composite. Our findings reveal that the 1:1 ratio exhibited the most favorable outcome (Figure S8c). In addition, the Co-MOF@rGO electrode’s oxidation–reduction peak distance is shortened (0.14–0.09 V). Due to the fact that rGO by itself did not store energy in this system (Fig. 5b), the nanocomposite sample possessed superior electrical conductivity and double-layer capacitance. Clearly, multicomponent MOF electrodes increase electrochemical efficacy.

Based on the redox potential of Co species, the following redox reactions are most likely involved in the charge storage processes of Co-MOF-71^42^:

2 $$\left[ {{\text{CoBDC}}} \right] + {\text{OH}}^{ - } - e^{ - } \to \left[ {{\text{CoBDC }}\left( {{\text{OH}}} \right)} \right]$$

3 $$\left[ {{\text{CoBDC }}\left( {{\text{OH}}} \right)} \right] \, + K^{ + } + e^{ - } \to {\text{ K}}\left[ {{\text{CoOH}}} \right]\left( {{\text{BDC}}} \right)$$

4 $${\text{K}}\left[ {{\text{CoOH}}} \right]\left( {{\text{BDC}}} \right) + {\text{OH}}^{ - } - e^{ - } \to {\text{K}}[{\text{Co}}\left( {{\text{OH}}} \right)_{2} ]\left( {{\text{BDC}}} \right)$$

5 $${\text{K}}[{\text{Co}}\left( {{\text{OH}}} \right)_{2} ]\left( {{\text{BDC}}} \right) + K^{ + } + e^{ - } \to {\text{Li}}_{2} {\text{BDC}} + {\text{Co}}\left( {{\text{OH}}} \right)_{2}$$

6 $${\text{Co}}\left( {{\text{OH}}} \right)_{2} + {\text{OH}}^{ - } - e^{ - } \cdot {\text{CoOOH}} + {\text{H}}_{2} {\text{O}}$$

It has been determined that ${\text{OH}}^{ - }$ ions are more nucleophilic than ${\text{BDC}}^{2 - }$ ligands. When ${\text{Co}}^{3 + }$ is reduced to ${\text{Co}}^{2 + }$ in electrochemical reactions, depicting in Eqs. (3) and (5), the –${\text{COO}}^{ - }$ of ${\text{BDC}}^{2 - }$ connected to the cobalt atom is removed instead of ${\text{OH}}^{ - }$. In addition, ${\text{OH}}^{ - }$ assaults the cobalt atom rather than the –${\text{COO}}^{ - }$ of ${\text{BDC}}^{2 - }$, oxidizing ${\text{Co}}^{2 + }$ to ${\text{Co}}^{3 + }$ in accordance with Eq. (4). Under the influence of an electric field, ${\text{OH}}^{ - }$ ions progressively replace ${\text{BDC}}^{2 - }$ ions in MOFs, which ultimately results in the transformation of Co-MOF into Co$\left( {{\text{OH}}} \right)_{2}$ and CoOOH^42^.

Furthermore, the redox reaction mechanism, which is a surface capacitance or diffusion-driven process, can be validated using the relationship between peak current $i_{p}$ and scan rate (mVs^−1^) from the CV curves. This relationship can be described by the equation $i_{p} = a{\text{V}}^{b}$, where b indicates the charge storage mechanism. The logarithm of the current ($i_{p}$) plotted against the logarithm of the voltage (υ) is employed in the determination of the magnitude of b. Diffusion-controlled or surface capacitance-dominated processes occur as the value approaches 0.5 or 1, respectively^64^. The b values for Co-MOF and Co-MOF@rGo electrodes were 0.66 and 0.62, respectively. This implies that charge storage reactions are controlled by diffusion (see Figure S9, Supporting Information, for details).

The findings of the Galvanostatic Charge Discharge (GCD) examination for the synthesised materials are depicted in Fig. 5c. The CO-MOF@rGO sample had a longer discharge time, indicating higher capacity. The GCD graphs (Fig. 5d) revealed that when the CO-MOF electrode was initially charged, the potential rose rapidly; however, electrochemical reactions may have caused the potential to rise more slowly over time. During the initial phase of the discharge procedure, a slight decrease in potential, commonly referred to as the IR drop, may arise due to factors such as electrolyte resistance, contact resistance between the active materials within the electrode, or series resistance (ESR) inherent in the system. This minimal IR demonstrates that the MOF nanohybrid electrode has superior rate capabilities and electrochemical contributions overall. The discharge process of the graph exhibits a gradual incline as a result of the electrochemical oxidation–reduction reactions. Subsequently, the plot exhibits a pronounced decrease in potential towards zero, demonstrating the Faraday characteristic of the material. The Co-MOF@rGO nanocomposite electrode had a specific capacity of 967.68 ${\text{Fg}}^{ - 1}$ at 1 A${\text{g}}^{ - 1}$, which was significantly higher than Co-MOF (651.75 ${\text{Fg}}^{ - 1}$ at 1 A${\text{g}}^{ - 1}$). It can be attributed to several factors, including the inherent characteristics of the nanosheet array, the presence of numerous active sites, the exceptional mass transfer qualities, and the synergistic effect resulting from the combination of Co and rGO.

As depicted in Fig. 5e, the progressive rise in current density leads to a gradual reduction in Cs values, owing to the influence of the internal resistance of the electrochemical system. Furthermore, the volumetric capacitance of materials was also computed and shown in Fig. 5f.

Electrochemical impedance spectroscopy (EIS) spectra were taken to investigate electrochemical processes at the electrode surface (Fig. 5g, h). Two frequency regions are represented in EIS profiles: a semicircle at high frequency, corresponding to faradic resistances and surface charge transfer ($R_{ct}$), and a straight line at low frequency, demonstrating ion diffusion resistances (Warburg impedances). In the high-frequency region, the connection point of the Nyquist plot and the real impedance axis is the equivalent series resistance (ESR), which consists of the inherent resistance of the electrode and electrolyte and the contact resistance between the current collector and the active material^65^. The Co-MOF@rGO sample had lower charge transfer ($R_{ct}$) and series resistance ($R_{s}$) than pristine samples. The presence of more active Co-MOF sites and rGO improved electron transfer at the electrode–electrolyte interface.

The EIS of the optimal supercapacitor electrode has an angle of approximately 45 degrees with respect to an imaginary axis^66^. As shown in Fig. 5h, Co-MOF and Co-MOF@rGO have a similar pattern. The constant phase element (CPE) model can explain nonuniform diffusion or multiple system routes^67^. The CPE consists of the alpha exponent’s pseudo-capacitance and roughness area, which rise as alpha decreases. Co-MOF@rGO has a higher admittance than Co-MOF, which results in increased pseudo-capacitance.

Co-MOF and Co-MOF@rGO electrode capacitance retention with cycle number is depicted in Fig. 5i. The Co-MOF@rGO electrode has a remarkable 95.4% stability. The synergistic effect of Co-MOF and rGO reduces mechanical deformation during cycling redox reactions, thereby enhancing cycle stability. In contrast, the Co-MOF electrode retains 80% of its capacitance and is less stable.

### Device fabrication

**Figure 6:**
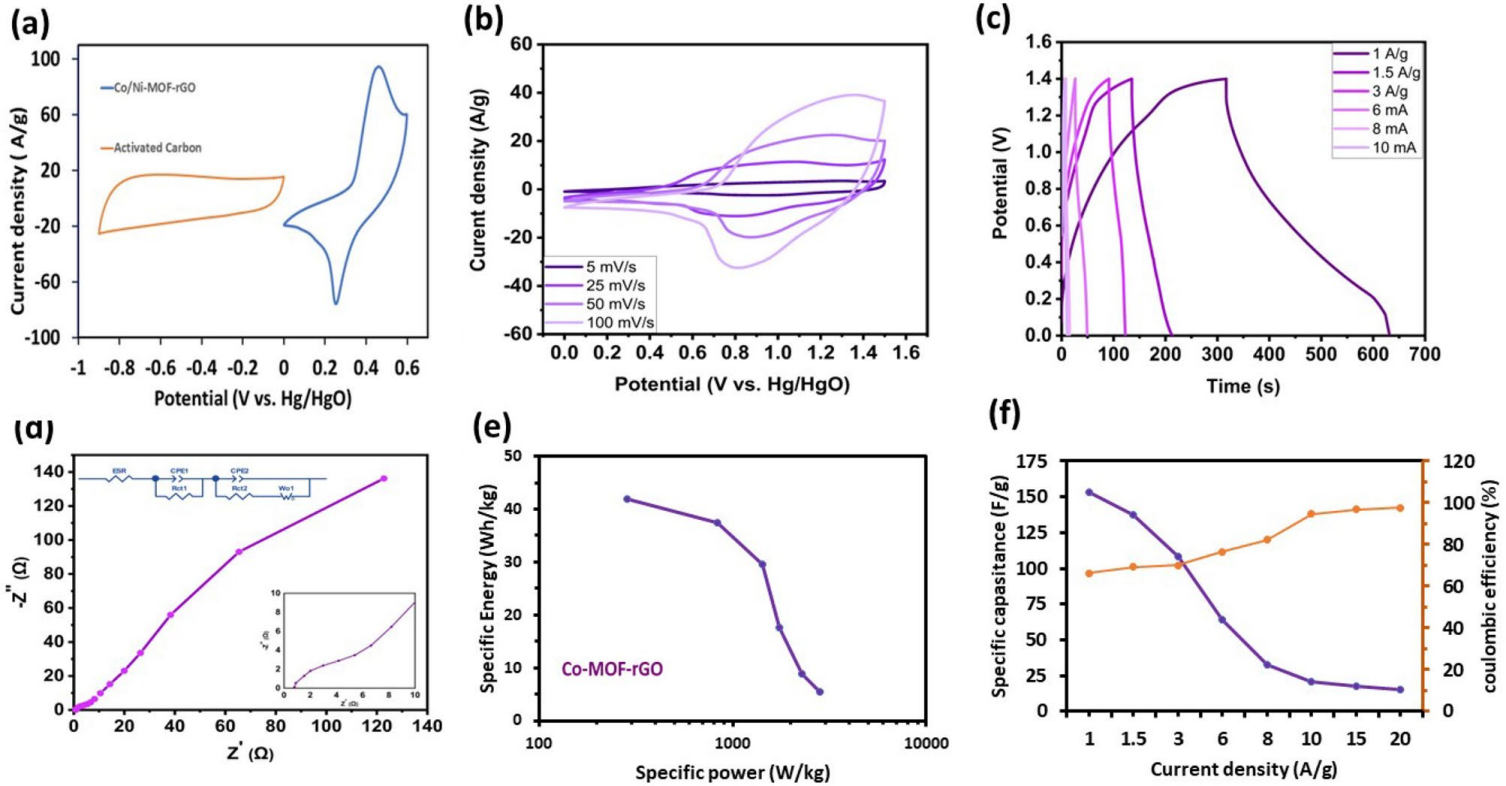
(**a**) CV of activated carbon as a negative electrode, Co-MOF-rGO as a positive electrode at a scan rate of 25 mV ${\text{s}}^{ - 1}$. (**b**) CVs of the Co-MOF-rGO||AC device at various scan rate of 5–100 mV ${\text{s}}^{ - 1}$. (**c**) GCD curves of the Co-MOF-rGO||AC device at different current densities from 1 to 10 A${\text{g}}^{ - 1}$. (**d**) Nyquist plot of Co/Ni-MOF@rGO||AC device. (**e**) Ragone plot-specific energy vs specific power of the device at different specific currents from 1 to 10 A${\text{g}}^{ - 1}$. (**f**) Rate capability plot of the Co-MOF@rGO||AC device at different specific currents from 1 to 20 A${\text{g}}^{ - 1}$ (primary y-axis). CE % at different rates (secondary y-axis). All the studies were performed in a 6.0 M KOH electrolyte.

An asymmetric supercapacitor was created to study the Co-MOF-rGO nanocomposite electrode’s potential applications. The positive electrode was composed of Co-MOF-rGO, whereas the negative electrode comprised activated carbon (AC). Figure 6a shows the CV curve of the positive and negative electrodes in a three-electrode cell configuration with a scanning speed of 25 mVs^−1^. Based on the shape of this curve, we can infer that the operating range of the AC electrode is between 0 and − 0.9 V, whereas the efficiency range of the Co-MOF-rGO nanocomposite electrode is between 0 and 0.6 V. As a result, the asymmetric device can function from 0 to 1.5 V. We tested the constructed asymmetric supercapacitor at various scanning speeds ranging from 5 to 100 mvs^−1^. With increased scanning speed, the nanocomposite-based supercapacitor maintains its form (Fig. 6b). The GCD profile of the supercapacitor device at a range of current densities is illustrated in Fig. 6c. The device maintains its triangular shape and exhibits a minimal infrared (IR) drop during the initial discharge phase, even at high rates. This suggests that the device possesses a significantly low equivalent series resistance (ESR). As is observed from the Nyquist plot (Fig. 6d), the effective series resistance (ESR) value is slightly over 1 Ω, which shows the low internal resistance of the device. The equivalent circuit fitted with EIS shows that the device has quick response kinetics and low charge transfer resistances at medium frequencies since it is composed of two overlapped semicircles with sizes of 0.15 and 8.9 Ohm. These values, together with the straight line in the low-frequency region, confirm the performance of the hybrid charge storage that includes the characteristics of the pseudo-capacitor and the Electric double-layer capacitors (EDLC). According to the Ragone diagrams of this device (Fig. 6e), at a current density of 1 A${\text{g}}^{ - 1}$, the specific capacitance of the asymmetric supercapacitor was 153.6 F${\text{g}}^{ - 1}$. This system provides high specific energy (41.82 Wh ${\text{g}}^{ - 1}$) and specific power (283.9 W ${\text{g}}^{ - 1}$) at a current density of 1 A${\text{g}}^{ - 1}$. The specific capacity versus specific current for the Co-MOF-rGO||AC device is seen in Fig. 6f. The specific capacity values exhibit a progressive reduction as the particular current increases. Nevertheless, the Coulombic efficiency (CE) tends to reach a value close to 100%. Hence, the utilisation of a single-step plasma synthesis method for the production of Co-MOF-rGO results in the creation of an electrode material exhibiting favourable characteristics such as high-power density and minimal energy density losses, rendering it well-suited for implementation in supercapacitor devices.

## Conclusion

The fabrication of Co-MOF-71 and Co-MOF-rGO supercapacitor electrodes was carried out under ambient conditions using a one-step cold plasma technique. The nanocomposite of Co-MOF@rGO preserves the nanosheet morphology observed in Co-MOF, with an average thickness of 20 nm. Hence, the utilisation of gas–liquid plasma synthesis has the capacity to modify the morphology and yield a nanostructure characterised by porosity and a three-dimensional configuration. The electrode containing Co-MOF@rGO demonstrated a Cs value of 967.68 Fg^−1^ when tested at 1 Ag^−1^, whereas the electrode containing Co-MOF exhibited a Cs value of 651.76 Fg^−1^ under the same conditions. In addition, the hybrid device consisting of Co-MOF@rGO||AC exhibits a notable specific energy of 41.82 Wh ${\text{kg}}^{ - 1}$ and a specific power reaching up to 284 W ${\text{kg}}^{ - 1}$. This one-pot synthesis method is extremely environmentally friendly because it does not require any pre- or post-treatments. We believe that gas–liquid plasma provides a rapid and safe process for the synthesis of nanocomposites with a unique structure that are ideal candidates for energy storage devices.

### Supplementary Information

Supplementary Information 1.

## Data Availability

The data that support the findings of this study are available within the article and its supplementary material.
