# Supplementary material for: Rapid cold plasma synthesis of cobalt metal–organic framework/reduced graphene oxide nanocomposites for use as supercapacitor electrodes
Source: Sci Rep. 2023 Sep 13;13:15156. doi: 10.1038/s41598-023-41816-9 (PMC10499990; doi:10.1038/s41598-023-41816-9)
Supplement: Supplementary file 1 — Supplementary Information 1. [file 41598_2023_41816_MOESM1_ESM.docx]

**Supplementary Information**

**Rapid cold plasma synthesis of cobalt metal-organic framework/reduced graphene oxide nanocomposites for use as supercapacitor electrodes**

**Zeinab Karimzadeh^1^, Babak Shokri^1,2^**^*^ **and Ali Morsali^3^**^*^

^1^Laser and Plasma Research Institute, Shahid Beheshti University, P.O. Box 1983969411, Tehran, Iran.

^2^Faculty of Physics, Shahid Beheshti University, P.O. Box 1983969411, Tehran, Iran.

^3^Department of Chemistry, Faculty of Sciences, Tarbiat Modares University, P.O. Box 14115-175, Tehran, Iran. ^*^ email: [b-shokri@sbu.ac.ir](mailto:b-shokri@sbu.ac.ir); [morsali_a@modares.ac.ir](mailto:morsali_a@modares.ac.ir)

**EXPERIMENTAL SECTION**

**Discharge parameters.** Using an oscilloscope, the applied voltage for the synthesis of MOFs with DBD plasma was measured at a peak-to-peak voltage of 6.0–7.0 kV. **Figure S1** depicts the voltage vs. time curves for three distinct voltages. During the synthesis process, the electrical behavior of the wave was nearly constant, indicating the stability of the electrical discharge.


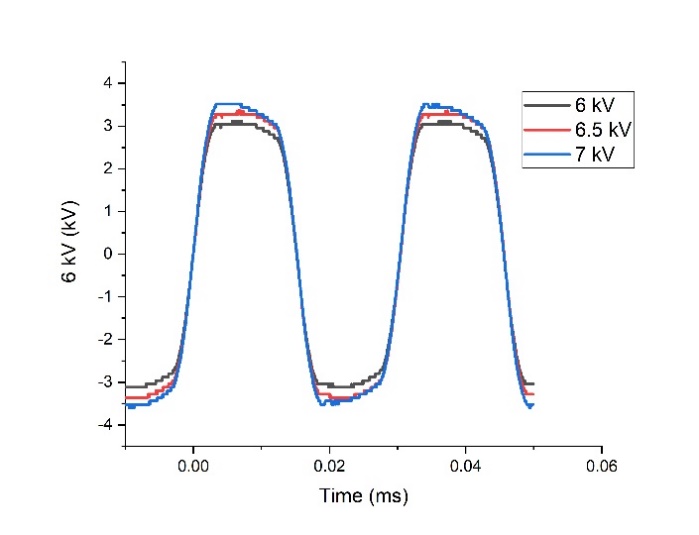


Figure S1. Voltage curves in different peaks to the peak.

**Optimization of MOFs Synthesis Condition.** For the plasma synthesis of MOFs and their composites, we considered three distinct parameters: discharge time, discharge voltage, and reactant mass ratio. In order to optimize the plasma synthesis conditions, three distinct discharge durations of 30, 45, and 60 minutes were applied with varying potentials of 6, 6.5, and 7 kV. Co-MOF synthesized at an applied potential of 6.5 V for 45 minutes has an XRD pattern most similar to that of MOF-71 (**Fig. S2**). Fortunately, these conditions also exhibited the most effective electrochemical analysis performance (**Fig. S3**).

Table S1. Synthesis conditions for MOFs and their composite preparation.

| Discharge time  (min) | Discharge voltage (V) | Reactant concentration (mM) | Co: rGO ratio |
| --- | --- | --- | --- |
| Changed | 6.5 | 1 | 1:1 |
| 45 | Changed | 1 | Changed |
| 45 | 6.5 | Changed | 1:1 |
| 45 | 6.5 | 1 |  |


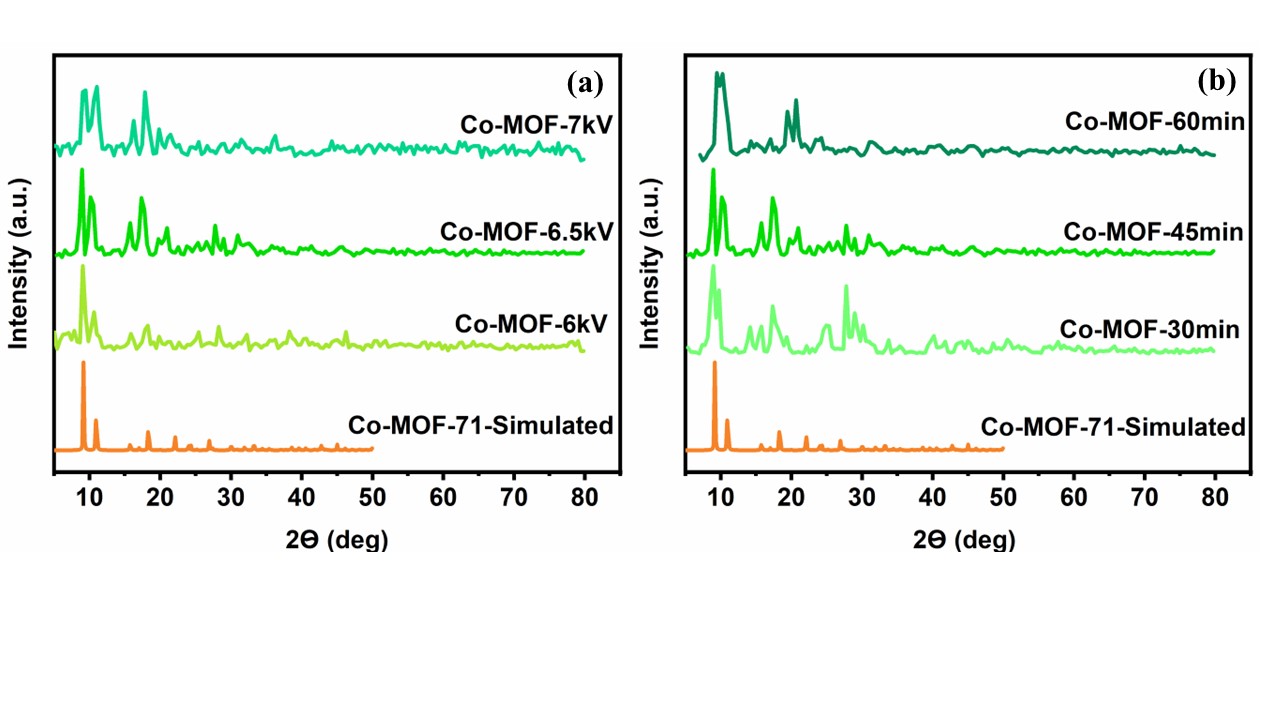
Figure S2. (a) XRD patterns of Co-MOF at different plasma DBD discharge voltages; (b) XRD patterns of Co-MOF at different plasma DBD discharge times.
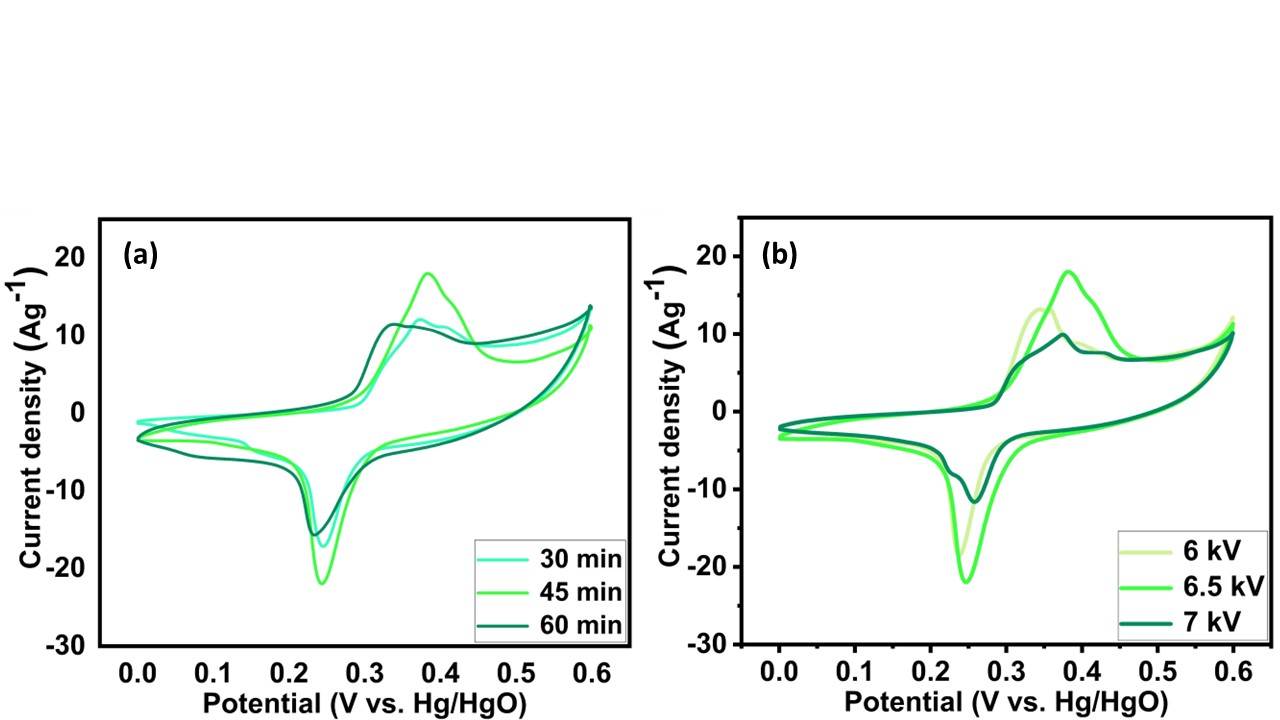


Figure S3. Optimization of the electrochemical synthesis conditions: The CV profiles of Co-MOF synthesis a) at various durations and b) at various voltages.

**Electrode preparation.** We combined 15 mg of active material (whether it's MOFs or nanocomposites) with 2.0 milligrams of polyvinylidene fluoride (PVDF) to serve as a binder and conductive additive, which was 2 mg of carbon black for making the positive electrode. The combination was stirred for 24 hours in N-methylpyrrolidone (NMP). The slurry was then applied to a current collector made of nickel foam (0.5 ${cm}^{2}$), and the electrode was allowed to dry at 60 ^∘^C for 24 hours. The mass loading of active material was approximately 2 ${mg cm}^{-2}.$

**Supercapacitor device fabrication.** In the asymmetric supercapacitor device fabrication, the positive electrode was Co/Ni-MOF@rGO/NF, the negative electrode was activated carbon (AC/NF), and the separator was non-woven fabrics (information on mass balance and other equations can be found in the **Supporting Information**, Calculations section).

**Electrochemical characterization.** In a three-electrode setup, electrochemical measurements were conducted using a CorrTest 350 electrochemical workstation (CorrTest Instruments Corp., Wuhan, China). As the working electrode, MOF/NF was utilized, while a platinum plate (1$\times$1 ${cm}^{2}$) and a Hg/HgO electrode served as the counter electrode and reference electrode, respectively. Cyclic voltammetry (CV), galvanostatic charge-discharge (GCD), and electrochemical impedance spectroscopy (EIS) experiments were utilized for measuring the electrochemical behaviour. CV testing was conducted in the potential range of 0–0.6 V at scan rates of 5, 25, 50, and 100 mV, and GCD studies were performed in the potential range of 0–0.5 V at current densities of 1, 2, 3, 5, 7, and 10 mA. The EIS measurements were conducted with a frequency range of 100 kHz to 0.1 Hz at the open-circuit potential. All measurements were performed at room temperature (25 °C) in an electrolyte containing 6 M KOH.

**Instrumentation.** Using X-ray diffraction analysis (XRD), the crystal structure of each sample was determined. XRD patterns were recorded on a Pw 1730 X-Ray Diffractometer (XRD, Philips X'pert diffractometer) using Cu K1 radiation (1.54056) and a 2-theta range of 5–80°. The X-ray tube's voltage and current are 40 kV and 30 mA, respectively. The FTIR spectra of the samples were acquired on a Thermo Nicolet infrared spectrometer (Avatar, USA) utilizing the potassium bromide (KBr) pellet technique. The samples' morphology was studied with a field-emission scanning electron microscope (FE-AEM, TESCAN MIRA3, Czech). As an adjunct to the SEM apparatus, an energy-dispersive X-ray spectrometer (EDX) was used to undertake elemental analyses. OES was detected by an Avantes fiber optic spectrometer (AvaSpec 3648-USB2, Netherlands) in the range of 200–1100 nm to detect the active species in the gas-liquid cold plasma.

**Calculation**

**Electrochemical calculation.** Utilizing the CV ($C_{s1}$) and GCD ($C_{s2}$ and $C_{s3}$) profiles, the following equations were used to calculate the specific capacitance (F $g^{-1}$) and specific capacity (mA h $g^{-1}$): ^1^

| $C_{s1}(F g^{-1})$*=*$\frac{\int I_{1}\mathrm{dV}}{v\times m\times\Delta V}$ | S1 |
| --- | --- |
| $C_{s2}(F g^{-1})$*=*$\frac{2I\int V\mathrm{dt}}{m{\times V}^{2}}$ | S2 |
| $C_{s3}(mA h g^{-1})=\frac{I\int V\mathrm{dt}}{3.6\times m\times V}$ | S3 |

where $C_{s}$ is the specific capacitance ($F g^{-1}$) or specific capacity ($mA h g^{-1}$), I (A) represents the discharge current, t (s) represents the discharge time, m (g) is the mass of the electrode materials, and V (V) represents the potential/voltage range in eq S2 and potential at the midpoint of the discharge step in eq S3. While $I_{1}$ (A) is the response current, Δ*V* (V) is the voltage window, and *v* (V$s^{-1}$) is the scan rate in eq S1.

The following formulas were used to compute the specific energy $E_{S}$ (W h k$g^{-1}$) and specific power, $P_{S}$, (W k$g^{-1}$) according to the overall mass of the active materials on both the positive and negative electrodes.

| $E_{S}(W h kg^{-1})=\frac{I\int V\mathrm{dt}}{3.6\times m}$ | S4 |
| --- | --- |
| $P_{S}(W kg^{-1})=\frac{3.6\times E_{S}}{t}$ | S5 |

The Coulombic efficiency was determined by using Equation (S7).

| Coulombic efficiency (η)= $\frac{t_{d}}{t_{c}}\times100$ | S6 |
| --- | --- |

where $t_{d}$ denotes the GCD curve's discharge time (s) and $t_{C}$ indicates the GCD curve's charging time (s).

**The loading determination for AC on coated NF.** By balancing the charges between the positive and negative electrodes, the mass loading of AC was established. S3 may be used to determine the optimal mass ratio between two electrodes. Where $m^{+}$ (g) is the mass loading, $C^{+}$ (F) and ${\Delta V}^{+}$ (V) denote, respectively, the specific capacitance and the potential window of the positive electrode. $m^{-}$, $C^{-}$and ${\Delta V}^{-}$ represent the negative electrode's mass loading, specific capacitance, and potential window, respectively.

| $\frac{m^{+}}{m^{-}}=\frac{C^{-}{\Delta V}^{-}}{C^{+}{\Delta V}^{+}}$ | S7 |
| --- | --- |

**Analytical characteristics**

Figure S4. SEM images of a) Co-MOF-6 kV, b) Co-MOF-6.5 kV, c) Co-MOF-7 kV, d) Solvothermal synthesis of Co-MOF. This sample was made using the hydrothermal method at a temperature of 120 degrees Celsius and a duration of 12 hours. Hydrothermally synthesized Co-MOF has a sedimentary and non-porous structure, as can be seen.


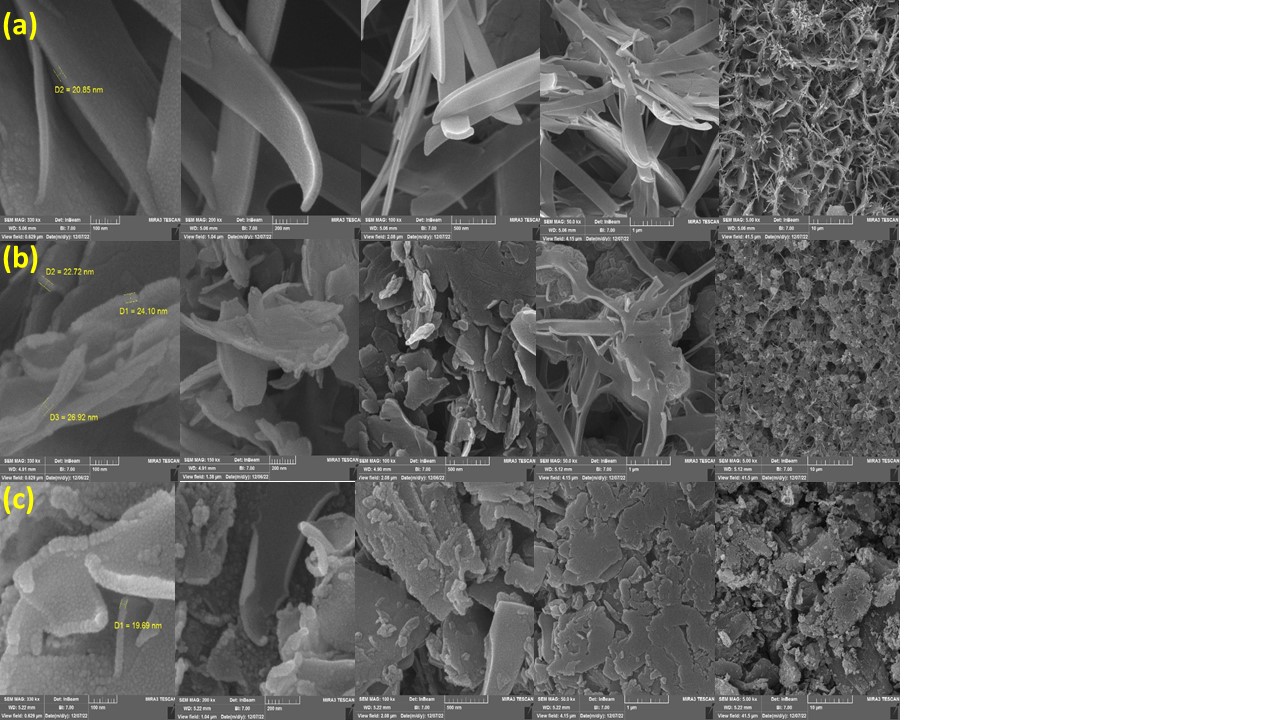


Figure S5. SEM images of a) Co-MOF-rGO (2:1), b) Co-MOF-rGO (1:1), c) Co-MOF-rGO (1:2)


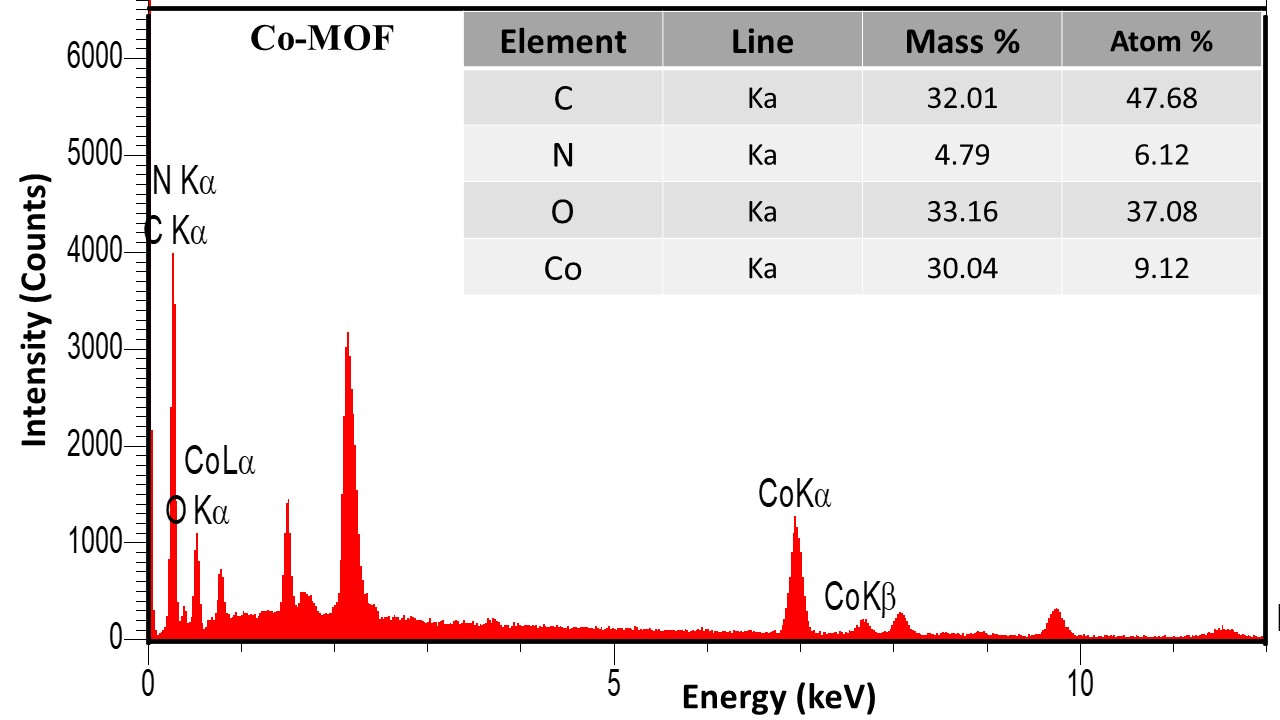


Figure S6. EDX analysis of Co-MOF


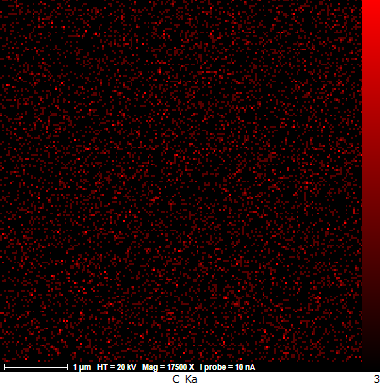

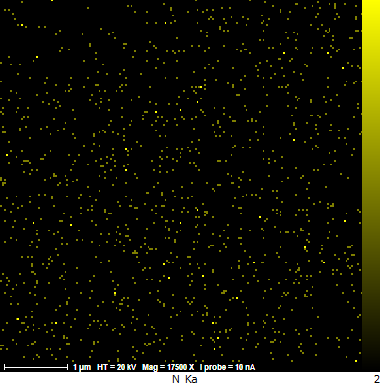

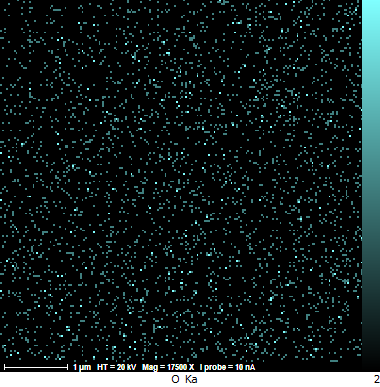

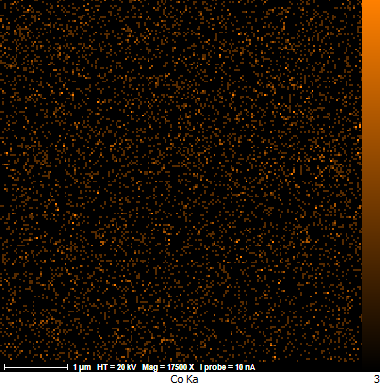

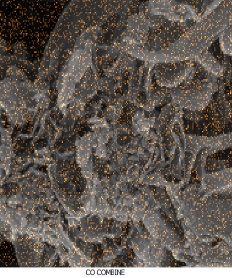


**(C)**

**(N)**

**(Co)**

**(O)**

Figure S7. EDX mapping of Co-MOF@rGO

| **BET** | **a_s,BET_**  **[m^2^ g^-1^]** | **Total-pore volume(p/p_0_=0.990)**  **[cm^3^ g^-1^]** | **Mean-pore diameter**  **[nm]** | **a_s,Lang_**  **[m^2^ g^-1^]** | **a_p(BJH)_**  **[m^2^ g^-1^]** | ***r_p,peak_*(*Area*)** |
| --- | --- | --- | --- | --- | --- | --- |
| **Co-MOF** | 52.543 | 0.3692 | 28.103 | 23158 | 64.008 | 6.06 |
| **Co-MOF-rGO** | 50.14 | 0.35 | 26.31 | 13684 | 59.92 | 2.09 |
| **Ref 10** | 10.43 | 0.025 | 9.773 | 20.50 |  |  |

Table S2. BET data of Co-MOF synthesis by gas-liquid plasma and Ref. 10

Table S3. Equivalent series resistance (**ESR**) and charge transfer resistance ($\boldsymbol{R}_{\boldsymbol{ct}}$) from Nyquist plots of all the synthesized materials

|  | ESR (Ω) | $R_{ct}$(Ω) |
| --- | --- | --- |
| **Co-MOF** | 1.27 | 6.84 |
| **Co-MOF@rGO** | 0.3 | 0.32 |


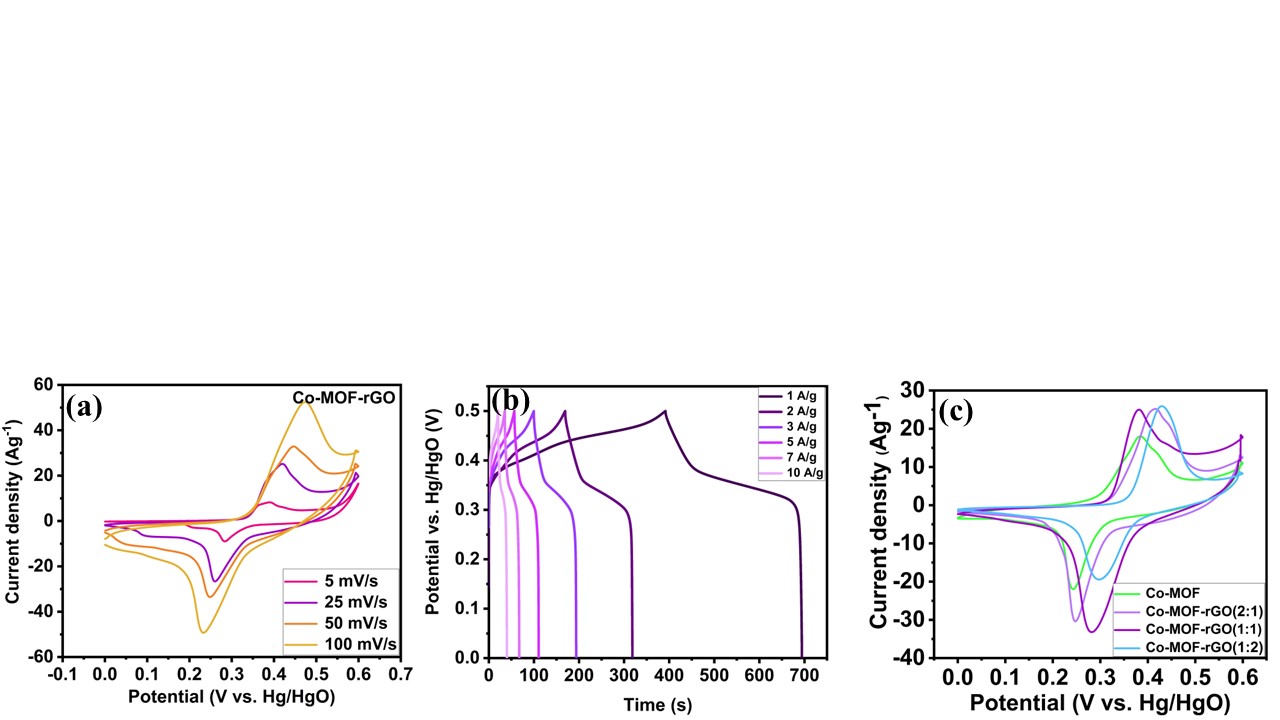


Figure S8. a) CVs of Co-MOF-rGO at different scan rates; b) GCD curves of the Co-MOF-rGO electrode at different current densities; C) CVs of Co-MOF-rGO composites at different ratios. All the CVs are recorded in a 6.0 M KOH electrolyte

**Contribution of Capacitive and Non-capacitive Faradaic Processes.** The current in the cyclic voltammogram has two parts: one controlled by capacitance and the other by diffusion. To determine the contribution of capacitive and diffusion-controlled processes to the total charge stored in the MOF, the anodic and cathodic peak current densities are plotted against the square root of the potential scan speed. The maximum current measured from the CV curves at different scan rates follows the power law equation:

| $i_{p}=aƲ^{b}$ | S7 |
| --- | --- |

where **a** and **b** are adjustable parameters. The **b** value changes between 0.5 and 1. When b = 0.5, the current has a linear relationship with the square root of the scan speed, and diffusion controls the charge storage process. Whenever b = 1, a capacitive process is primarily responsible for charging. The slope of the log ($i_{p}$) versus log(Ʋ) graph (**Figure S9a**) gives the value of b, which is 0.46 for the Co-MOF electrode. This figure shows that the peak current and the scan rate have a linear relationship, which indicates that the charge is mainly controlled by a diffusion process. In addition, by drawing the curve in terms of Ʋ and $Ʋ^{1/2}$ (**Figure S9C**), it can be seen that the current changes linearly with both components. Therefore, in the Co-MOF sample, the current can be a combination of capacitive and diffusion-controlled charge storage processes. To calculate the amount of load from the diffusion-controlled process, we assume that the total load is the sum of the loads resulting from capacitive processes $k_{1}Ʋ$ and diffusion-controlled processes ($k_{2}Ʋ^{1/2})$. Therefore, the power law equation can be written as follows:

| $i_{p}=k_{1}Ʋ+k_{2}Ʋ^{1/2}$ | S8 |
| --- | --- |

And,

| ${i_{p}}/{Ʋ^{1/2}}=k_{1}Ʋ^{1/2}+k_{2}$ | S9 |
| --- | --- |

where $i_{p}$ is the cathodic peak current and $k_{1}Ʋ$ and $k_{2}Ʋ^{1/2}$ is the current contribution of capacitive and non-capacitive processes. $k_{1}$ and $k_{2}$ are respectively obtained from the slope and the extrapolation of the curve ${i_{p}}/{Ʋ^{1/2}}-Ʋ^{1/2}$ . The amounts of $k_{1}$ and $k_{2}$for Co-MOF are equal to 21% and 9% while for Co-MOF-rGO are equal to 27% and 10%. The high contribution of $k_{1}$ from the sum of $k_{1}+k_{2}$indicates the high impact of the diffusion phenomenon in electrochemical performance and charge storage with diffusion processes in the overall charging.


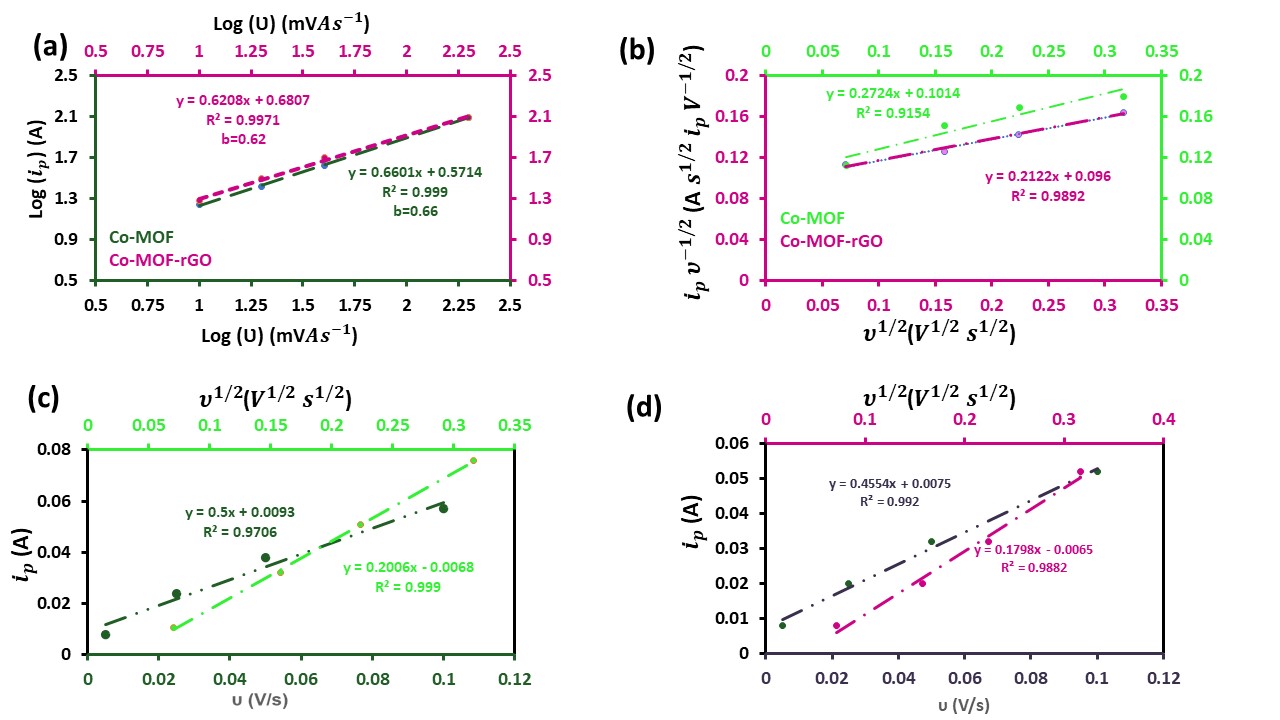


Figure S9.(a) Plot of log ($i_{p}$) vs log (Ʋ) for Co-MOF and Co-MOF-rGO; (b) plot of ${i_{p}}/{Ʋ^{1/2}}vs Ʋ^{1/2}derived from the CVs$; (c) plots of $i_{p}$ vs Ʋ and $Ʋ^{1/2}$ for Co-MOF and (d) plots of $i_{p}$ vs Ʋ and $Ʋ^{1/2}$ for Co-MOF-rGO


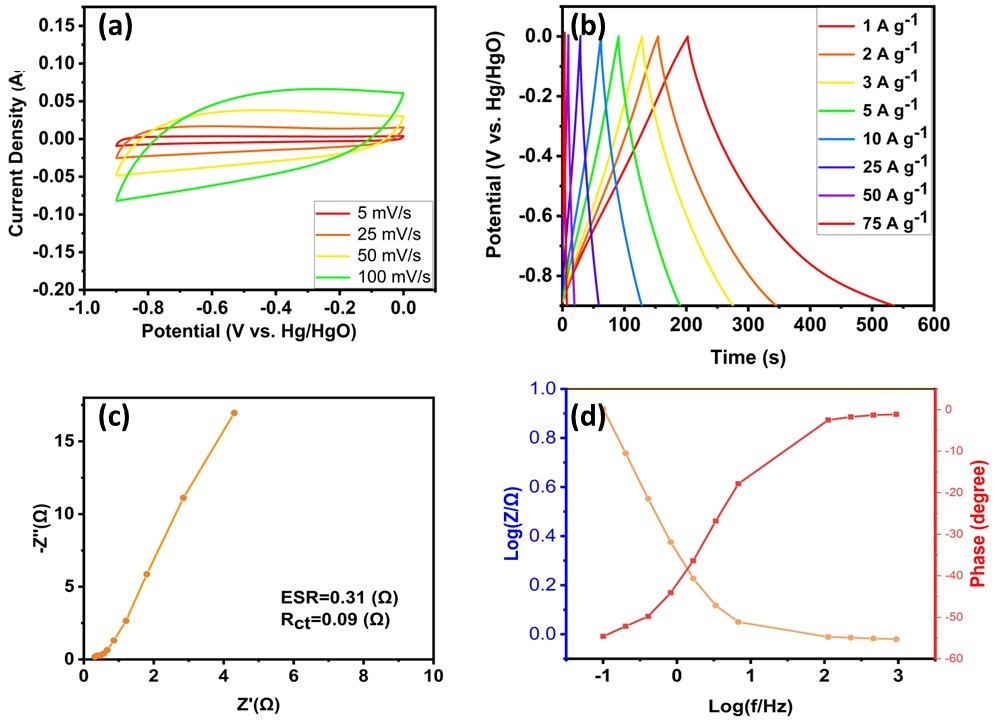
**Electrochemical characterization of the AC negative electrode**

Figure S10. (a) CV curves of the AC electrode at different scan rates from 5 to 100 mV s-1. (b) GCD profiles of the AC electrode at different current density values from 1 to 150 A g-1. (c) Nyquist plot and (d) Bode phase and magnitude plots of the AC electrode in the frequency range from 100 kHz to 100 mHz at an open circuit potential (OCP).

*Table S4. Comparison of the Energy Storage Performances of Co-MOF in Both 3E- and 2E-cell Setups with Several Similar Materials and Devices Reported in the Literature*

| name | 3E Capacity/ Capacitance (F$\boldsymbol{g}^{\boldsymbol{-1}}$) | 2E Capacity/ Capacitance (F$\boldsymbol{g}^{\boldsymbol{-1}}$) | Specific Energy (Wh k$\boldsymbol{g}^{\boldsymbol{-1}}$) | Specific Power (W k$\boldsymbol{g}^{\boldsymbol{-1}}$) | Stability % (cycle number) | Synthesis method | Electrolyte | REF |
| --- | --- | --- | --- | --- | --- | --- | --- | --- |
| Co-MOF | 206 (0.6 A$g^{-1}$) |  |  |  | 98.5(1000) | hydrothermal | 1M LiOH | ^2^ |
| Co-BDC MOF | 131.8 (10 mV $s^{-1}$) |  | 20.7 | 3880 W k$g^{-1}$ | 94.3% (1000) | solvothermal | 0.5 M LiOH | ^3^ |
| Co-BTC nanoblock microspheres (CTNBMs) | 427.8(0.5 A $g^{-1}$) | 95.6(1A$g^{-1}$) | 30.6 | 349.7 | 85.9% (3000) | hydrothermal | 1 M NaOH | ^4^ |
| Co-MOF/NF | 711 |  |  |  |  | hydrothermal | 6 M KOH | ^5^ |
| Co-MOF | 220(1A$g^{-1}$) |  |  |  |  | hydrothermal | 6 M KOH | ^5^ |
| Co-MOF powder | 234 |  |  |  |  | hydrothermal | 2 M KOH | ^6^ |
| MOF-71 | 246 |  |  |  |  | solvothermal | 6 M KOH | ^7^ |
| Co-MOF | **651.7** |  | **23.3** | **3180** | **80% (4000)** | **Gas-liquid Plasma synthesis** | **6 M KOH** | **This Work** |
| Co-MOF-rGO | **967.7** |  | **34.6** | **4080** | **95% (4000)** | **Gas-liquid Plasma synthesis** | **6 M KOH** | **This Work** |

**Reference**

1. Noori, A., El-Kady, M. F., Rahmanifar, M. S., Kaner, R. B. & Mousavi, M. F. Towards establishing standard performance metrics for batteries, supercapacitors and beyond. *Chemical Society Reviews* vol. 48 1272–1341 at https://doi.org/10.1039/c8cs00581h (2019).

2. Lee, D. Y. *et al.* Unusual energy storage and charge retention in Co-based metal-organic-frameworks. *Microporous Mesoporous Mater.* **153**, 163–165 (2012).

3. Lee, D. Y. *et al.* Supercapacitive property of metal-organic-frameworks with different pore dimensions and morphology. *Microporous Mesoporous Mater.* **171**, 53–57 (2013).

4. Zhang, H. *et al.* Influence of Co-MOF morphological modulation on its electrochemical performance. *J. Phys. Chem. Solids* **160**, 110336 (2022).

5. Wang, J. *et al.* Rational construction of triangle-like nickel-cobalt bimetallic metal-organic framework nanosheets arrays as battery-type electrodes for hybrid supercapacitors. *J. Colloid Interface Sci.* **555**, 42–52 (2019).

6. Zhu, G. *et al.* A self-supported hierarchical Co-MOF as a supercapacitor electrode with ultrahigh areal capacitance and excellent rate performance. *Chem. Commun.* **54**, 10499–10502 (2018).

7. Bigdeli, H., Moradi, M., Hajati, S., Kiani, M. A. & Toth, J. Cobalt terephthalate MOF-templated synthesis of porous nano-crystalline Co3O4 by the new indirect solid state thermolysis as cathode material of asymmetric supercapacitor. *Phys. E Low-Dimensional Syst. Nanostructures* **94**, 158–166 (2017).
